# Interpretable Deep-Learning Approaches for Osteoporosis Risk Screening and Individualized Feature Analysis Using Large Population-Based Data: Model Development and Performance Evaluation

**DOI:** 10.2196/40179

**Published:** 2023-01-13

**Authors:** Bogyeong Suh, Heejin Yu, Hyeyeon Kim, Sanghwa Lee, Sunghye Kong, Jin-Woo Kim, Jongeun Choi

**Affiliations:** 1 School of Mechanical Engineering Yonsei University Seoul Republic of Korea; 2 Department of Family Medicine School of Medicine Ewha Womans University Seoul Republic of Korea; 3 Department of Internal Medicine Seoul National University Bundang Hospital Seongnam Republic of Korea; 4 Department of Oral and Maxillofacial Surgery School of Medicine Ewha Womans University Seoul Republic of Korea

**Keywords:** osteoporosis, artificial intelligence, deep learning, machine learning, risk factors, screening

## Abstract

**Background:**

Osteoporosis is one of the diseases that requires early screening and detection for its management. Common clinical tools and machine-learning (ML) models for screening osteoporosis have been developed, but they show limitations such as low accuracy. Moreover, these methods are confined to limited risk factors and lack individualized explanation.

**Objective:**

The aim of this study was to develop an interpretable deep-learning (DL) model for osteoporosis risk screening with clinical features. Clinical interpretation with individual explanations of feature contributions is provided using an explainable artificial intelligence (XAI) technique.

**Methods:**

We used two separate data sets: the National Health and Nutrition Examination Survey data sets from the United States (NHANES) and South Korea (KNHANES) with 8274 and 8680 respondents, respectively. The study population was classified according to the T-score of bone mineral density at the femoral neck or total femur. A DL model for osteoporosis diagnosis was trained on the data sets and significant risk factors were investigated with local interpretable model-agnostic explanations (LIME). The performance of the DL model was compared with that of ML models and conventional clinical tools. Additionally, contribution ranking of risk factors and individualized explanation of feature contribution were examined.

**Results:**

Our DL model showed area under the curve (AUC) values of 0.851 (95% CI 0.844-0.858) and 0.922 (95% CI 0.916-0.928) for the femoral neck and total femur bone mineral density, respectively, using the NHANES data set. The corresponding AUC values for the KNHANES data set were 0.827 (95% CI 0.821-0.833) and 0.912 (95% CI 0.898-0.927), respectively. Through the LIME method, significant features were induced, and each feature’s integrated contribution and interpretation for individual risk were determined.

**Conclusions:**

The developed DL model significantly outperforms conventional ML models and clinical tools. Our XAI model produces high-ranked features along with the integrated contributions of each feature, which facilitates the interpretation of individual risk. In summary, our interpretable model for osteoporosis risk screening outperformed state-of-the-art methods.

## Introduction

Osteoporosis is a skeletal disorder characterized by the loss of bone mass, microarchitectural deterioration of the bone tissue, and decline in bone quality, which lead to increased bone fragility and risk of fractures [1]. The prevalence of osteoporosis among adults aged 50 years and over is 12.6% in the United States and is higher among women (19.6%) than men (4.4%) [2]. The number of adults aged 50 years and older with osteoporosis has increased from 10.2 million in 2010 to 12.3 million in 2020 and is expected to reach 13.6 million in 2030 [3], indicating a gradual increase in the societal burden of this disease. International osteoporosis foundations report that approximately one-third of women and one-fifth of men aged 50 years or more experience osteoporotic fractures [4,5]. Osteoporotic fractures, particularly hip fractures, are associated with limited ambulation, chronic pain and disability, loss of independence, and decreased quality of life. Additionally, approximately 20% to 30% of patients with osteoporosis die within 1 year of experiencing osteoporotic fractures. Because osteoporosis is typically asymptomatic until a fracture occurs, early screening and detection are crucial strategies for osteoporosis management.

Dual-energy X-ray absorptiometry (DXA), particularly central DXA of the hip and lumbar spine, is currently the gold standard for measuring bone mineral density (BMD) to define osteoporosis. However, central DXA is not widely used due to its low availability and high cost [6]. Furthermore, self-demand based on the absence of symptoms until osteoporotic fracture has contributed to its limited utilization [6]. The annual DXA testing rate for elderly women covered by Medicare in the United States was approximately 14% from 2006 to 2010 [7] and overall screening rates were only 21.2% and 26.5% in women aged 50-64 and 65-79 years, respectively [8].

As another approach to screening, clinical assessment tools such as the Fracture Risk Assessment Tool (FRAX), Simple Calculated Osteoporosis Risk Estimation (SCORE), Osteoporosis Risk Assessment Instrument (ORAI), Osteoporosis Index of Risk (OSIRIS), and Osteoporosis Self-assessment Tool (OST) have been developed to identify patients at increased risk of osteoporosis. The pooled area under the curve (AUC) for these tools ranges from 0.65 to 0.70. FRAX, which is an extensively studied tool for predicting fracture risk, exhibits similar performance with an AUC ranging from 0.58 to 0.82 [9]. Despite their usefulness and convenience, these tools are applied on a limited basis due to their limited accuracy [10].

With an exponential increase in computing power in the big data era, machine-learning (ML) approaches have been rapidly adopted in the medical field, including in the diagnosis of bone diseases. Compared to the existing clinical tools, an artificial intelligence–based method has the advantage of analyzing diverse features in intertwined relationships with osteoporosis, resulting in higher accuracy. Several studies have shed light on ML approaches to osteoporosis diagnosis and detection, fracture prediction [11-13], and especially for medical imaging. Various studies have used ML methods for risk assessment based on medical databases. However, these early attempts revealed several limitations, including overfitting, lack of representativeness of a population, inappropriate validation using k-fold cross-validation, lack of confidence intervals around point estimates, and arbitrary variable selections [11,14-17]. Furthermore, insufficient accuracy, which is sometimes lower than that of conventional clinical assessment tools, hinders the widespread application of ML for osteoporosis risk prediction.

Therefore, we developed a deep learning (DL) model to screen osteoporosis risk and utilized explainable artificial intelligence (XAI) techniques to provide a clinical interpretation of the results from our model. To demonstrate the performance of our approach, we compared the results of our DL model to those of ML models and conventional clinical tools. We also demonstrate that our model provides individual explanations for feature contributions. To the best of our knowledge, this is the first study to use a DL approach for risk screening based on large population databases. Our model investigates the complex nonlinear relationships of variables that are not identifiable by conventional statistics using arbitrary feature engineering. Additionally, a comprehensive approach for individualized risk assessment and treatment decisions is proposed by considering various combinations of risk variables and complex aspects of diseases.

## Methods

### Study Design and Participants

This study utilized two cross-sectional data sets, namely the National Health and Nutrition Examination Survey data sets of the United States (NHANES) and South Korea (KNHANES). NHANES is a cross-sectional study conducted by the National Center for Health Statistics to assess the overall health and nutritional status of the population in the United States [18].

Four cycles of NHANES from 2005 to 2006, 2007 to 2008, 2009 to 2010, and 2013 to 2014 were used, and we incorporated only respondents over the age of 50 years or those who had gone through menopause. Two cycles of NHANES (from 2011 to 2012 and from 2015 to 2016) were excluded because femoral neck BMD and total femur BMD data were not available for those two cycles. In NHANES, the total femur BMD and femoral neck BMD were measured using DXA, which was performed using a Hologic QDR-4500A fan-beam densitometer (Hologic, Inc; Bedford, MA, USA).

KNHANES is a cross-sectional survey of South Korean health and nutrition statuses with a representative population set derived through sampling that has been conducted by the Korean Centers for Disease Control and Prevention (KCDC) since 1998 [19]. KNHANES data from 2008 to 2011 were utilized in this study. The data sets from 2008 to 2009 consisted of 9200 households and the data sets from 2010 to 2011 consisted of 7680 households. In KNHANES, femoral neck BMD and total femur BMD were investigated in respondents aged over 50 years or among those who had gone through menopause, which were measured using DXA performed with a Hologic DISCOVERY QDR-4500W device (Hologic, Inc).

### Ethics Approval

The methods were performed in accordance with relevant guidelines and regulations and approved by the Research Ethics Review Board of the National Center for Health Statistics (Protocol #2005-06, Protocol #2011-17). NHANES has obtained written informed consent from all respondents. KNHANES was approved by the KCDC Institutional Review Board (2008-04EXP-01-C, 2009-01CON-03-2C, 2010-02CON-21-C, 2011-02CON-06-C) and written consent was obtained from all participants.

### Assessment of Osteoporosis

For both NHANES and KNHANES, two different criteria based on the femoral neck BMD and total femur BMD were used to classify respondents into osteoporosis, osteopenia, and normal groups. Respondents with a T-score of femoral neck BMD or total femur BMD under −2.5 were defined as having osteoporosis. Those with T-scores between −1.0 and −2.5 were classified into the osteopenia group. The group with T-scores above −1.0 was defined as normal. For KNHANES, the T-scores were calculated using a reference group of Japanese individuals aged over 20 years [20] separately for the femoral neck and total femur. In the case of NHANES, we calculated the T-scores of respondents using a reference group consisting of non-Hispanic white women aged 20 to 29 years from NHANES III according to World Health Organization recommendations [21]. Using the calculated T-scores, we classified respondents into three groups: osteoporosis, osteopenia, and normal.

### Data Preprocessing

We only included respondents who had femoral neck and total femoral BMD records. Additionally, multiple variables with relevant information were merged into single variables or all but one variable were deleted. For example, nine variables indicating signs and symptoms of depression used in the Patient Health Questionnaire-9 (PHQ-9) [22] in NHANES were combined into one variable according to the PHQ-9 criteria. Multiple variables related to smoking, drinking, and blood pressure were eliminated, and we included only representative variables, as discussed previously [23,24]. Additionally, several variables with the same measurements but different units were removed, leaving only one variable. For KNHANES, several variables related to smoking, income, education level, drinking, and diabetes were merged or eliminated, following the data preprocessing methods described previously [25].

**Figure 1:**
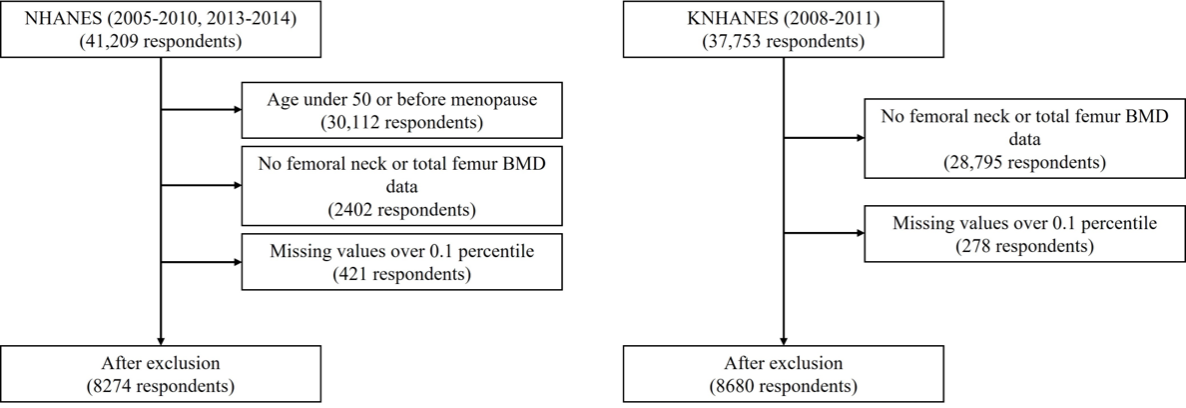
Flow diagram of overall data preprocessing. BMD: bone mineral density: KNHANES: Korean Health and Nutrition Examination Survey; NHANES: National Health and Nutrition Examination Survey (United States).

After merging variables, we excluded variables with nonnumeric values and considered responses of “refused” or “do not know” as missing values. Respondents who had missing values for more than 10% of all variables or variables that had missing values for more than 10% of all respondents were excluded. Missing values were filled in using k-nearest neighbors (KNN) imputation. At the end of preprocessing, a total of four types of data sets were created: two from NHANES and two from KNHANES, each using femoral neck BMD or total femur BMD when categorizing respondents into osteoporosis, osteopenia, or normal groups. The two data sets from NHANES comprised 8274 respondents with 89 variables and those from KNHANES comprised 8680 respondents with 162 variables. A flow diagram of the overall data processing is presented in Figure 1.

### Model Training Details

We implemented a DL algorithm and compared the results to those of ML algorithms. The DL model we used consisted of three layers: two dense layers with rectified linear unit activation functions and a final layer with a softmax activation function. Batch normalization and dropout layers were included after the two dense layers. Through five-fold cross-validation, the hyperparameters of the DL models were optimized based on five subsets of the created data sets. The optimized models for NHANES had two dense layers (each with 128 and 16 nodes for the femoral neck, and 128 and 64 nodes for the total femur) with a dropout rate of 0.2 using the Adam optimizer with a 0.005 learning rate. The models for KNHANES had two dense layers (each with 128 and 16 nodes for the femoral neck, and 128 and 32 nodes for the total femur) and used dropout rates of 0.2 (for femoral neck) and 0.4 (for total femur) with the Adam optimizer and a 0.005 learning rate. The models were trained and optimized in Keras using a TensorFlow backend (Google Inc) [26]. For the ML methods, seven models were considered for comparison: a nonlinear support vector machine, decision trees, extra trees, light gradient boosting machine (LGBM) classifier, logistic regression, KNN, and multilayer perceptron (MLP). We set the hyperparameters of the ML models to the default values within the scikit-learn package [27].

### Feature Contribution Ranking Analysis

After training the DL and ML models, we applied an algorithm to explain the DL model’s behavior and rank the features in order of importance. For the DL models, local interpretable model-agnostic explanations (LIME) [28] was adopted as an XAI technique. LIME is an algorithm that explains classifier predictions by perturbing inputs and performing searches using local interpretability. The output of LIME represents the relative importance of the variables for classification. Therefore, we analyzed the results in two manners. First, we added the resulting values from LIME for each variable and ranked contributions according to their values. This rank represents the order of the variables based on their impact on the DL model’s classification of osteoporosis. Second, we observed the positive or negative effects of each feature on the probability of osteoporosis with respect to the numerical or categorical values of the features.

For a similar reason, the Boruta [29] and least absolute shrinkage and selection operator (LASSO) [30] methods were applied to the ML model with the best performance to calculate the importance values of features. We utilized the measured relative feature importance from Boruta and coefficients from the output of LASSO to rank the contributions of features. Boruta was used with the 80th percentile of shadow feature importance and LASSO was used with an α value of 0.001, which is the weight given to the sum of the squares of coefficients.

### Clinical Osteoporosis Assessment Tools

Widely known clinical assessment tools for diagnosing osteoporosis, such as OST, ORAI, and OSIRIS, were used for comparisons to evaluate the performance of the DL models. We compared the performance of our DL model with that of the clinical osteoporosis assessment tools on both the NHANES and KNHANES data sets. When classifying osteoporosis, OST was used for both men and women, whereas ORAI and OSIRIS were applied only to the female data set.

### Statistical Analysis

Comparisons of the continuous variables from the NHANES and KNHANES data sets were performed using the Student *t*-test and are presented as mean (SD) values. Categorical variables were compared using the *χ*^2^ test and are presented as counts and percentages. A *P* value less than or equal to .001 was considered statistically significant. CIs were computed using the Student *t*-distribution.

## Results

### Baseline Characteristics

The baseline characteristics of the respondents from the preprocessed data sets are presented in Table 1. Among the 8274 respondents in NHANES, the average age was approximately 65 years and 52% were men. The KNHANES respondents exhibited a similar distribution with a male ratio of 45% and average age of approximately 64 years. For both NHANES and KNHANES, more respondents were classified into the osteoporosis or osteopenia groups when femoral neck BMD was used instead of total femur BMD.

**Table 1:** General characteristics of respondents.

Variable		NHANES^a^ (N=8274)	KNHANES^b^ (N=8680)	*P* value^c^	
**Sex, n (%)**					<.001
	Male	4283 (51.76)	3899 (44.92)		
	Female	3991 (48.24)	4781 (55.08)		
Age (years), mean (SD)		64.89 (9.67)	63.83 (8.99)	<.001	
**Economic status, mean (SD)**					<.001
	Family PIR^d^	2.71 (1.54)	N/A^e^		
	Household income	N/A	141.07 (134.24)		
**Drinking, n (%)**					<.001
	At least 12 drinks/year	5705 (68.95)	N/A		
	High-risk drinker	N/A	682 (7.86)		
Smoked at least 100 cigarettes in lifetime, n (%)		4316 (52.16)	3475 (40.03)	<.001	
No physical ability limitation, n (%)		7269 (87.85)	6465 (74.48)	<.001	
BMI (kg/m^2^), mean (SD)		28.46 (5.47)	23.94 (3.13)	<.001	
Diabetes, n (%)		1457 (17.61)	1528 (17.61)	.05	
Hypertension, n (%)		4344 (52.50)	4529 (52.18)	<.001	
Depression, n (%)		532 (6.43)	437 (5.03)	<.001	
**Bone mineral density (g/cm^2^), mean (SD)**					<.001
	Femoral neck	0.77 (0.14)	0.67 (0.13)		
	Total femur	0.93 (0.17)	0.84 (0.15)		
Osteoporosis family history, n (%)		1031 (12.456)	1252 (14.42)	<.001	
**Osteoporosis–femoral neck, n (%)**					<.001
	Normal	4564 (55.16)	4204 (48.43)		
	Osteopenia	3233 (39.07)	3255 (37.50)		
	Osteoporosis	477 (5.77)	1221 (14.07)		
**Osteoporosis–total femur, n (%)**					<.001
	Normal	6031 (72.89)	6095 (70.22)		
	Osteopenia	1965 (23.75)	2313 (26.65)		
	Osteoporosis	278 (3.36)	272 (3.13)		

^a^NHANES: US National Health and Nutrition Examination Survey.

^b^KNHANES: Korean National Health and Nutrition Examination Survey.

^c^*P* values for continuous variables are calculated with one-way analysis of variance and those for categorical variables are calculated from the *χ*^2^ test.

^d^PIR: poverty income ratio.

^e^N/A: not applicable.

### Performance Comparisons

**Figure 2:**
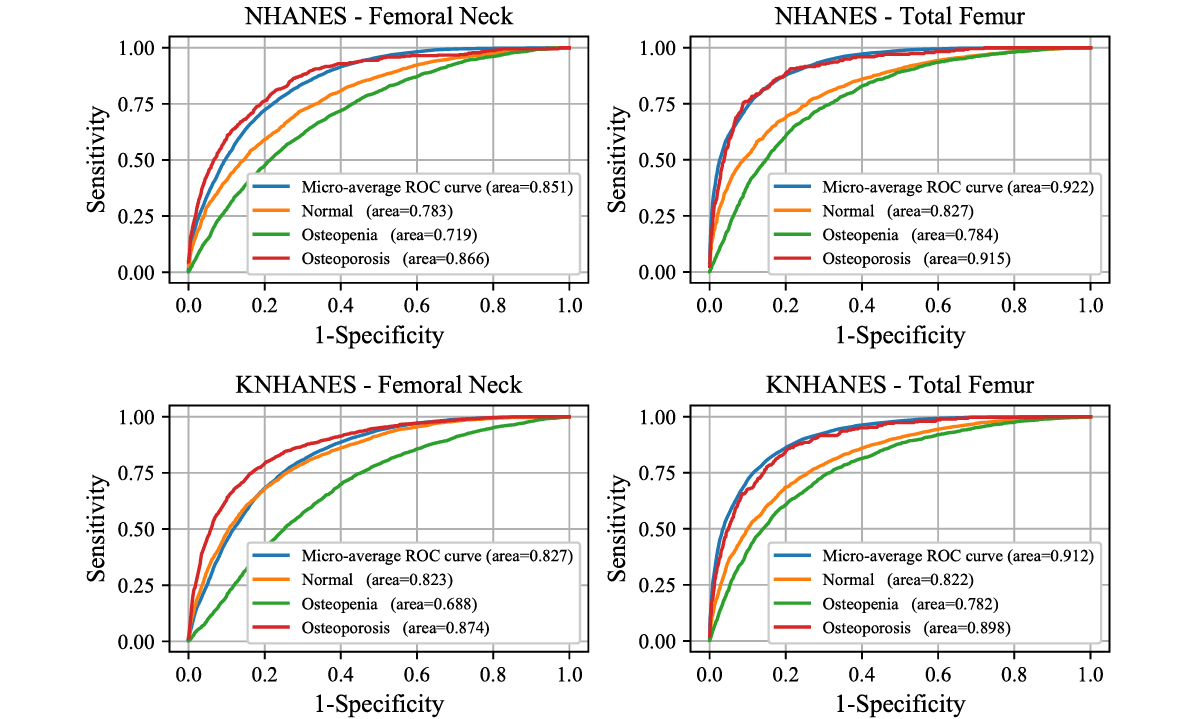
Performance of deep-learning (DL) models on the NHANES and KNHANES data sets. Receiver operating characteristic (ROC) curves of the DL models applied to NHANES and KNHANES femoral neck and total femur bone mineral density (BMD). The area under the curve values of individual classes are noted in the graphs. KNANES: Korean National Health and Nutrition Examination Survey; NHANES: US National Health and Nutrition Examination Survey.

**Figure 3:**
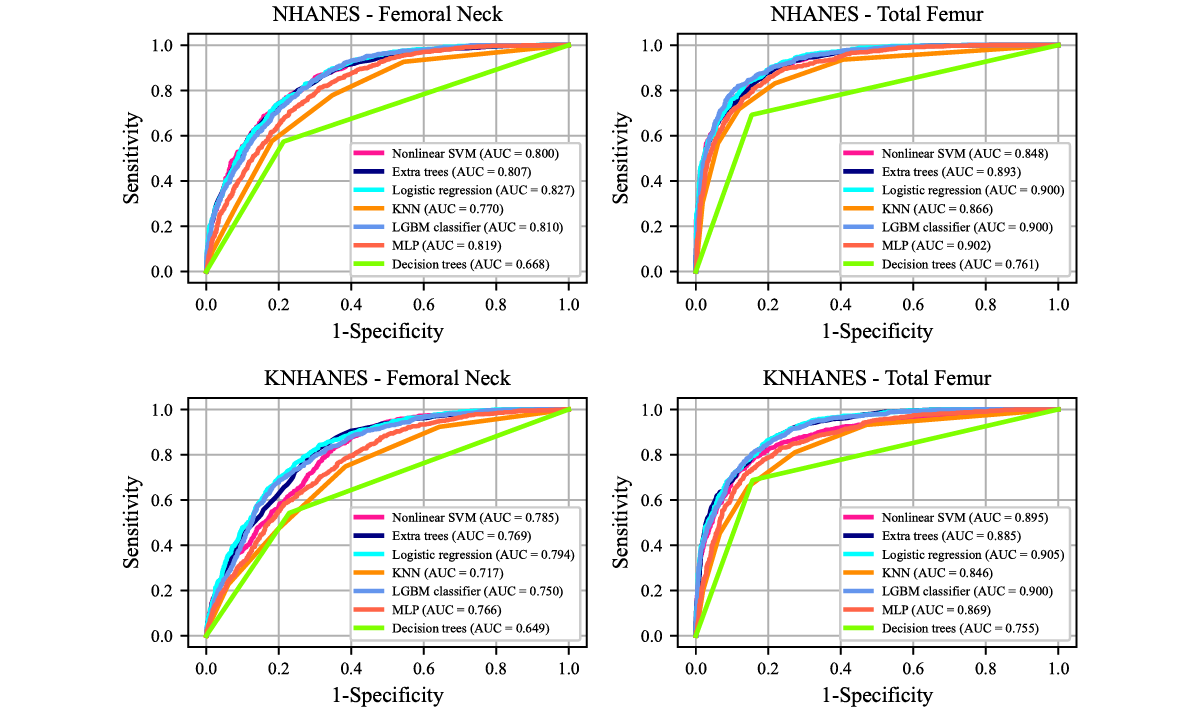
Performance of machine-learning (ML) models on the NHANES and KNHANES data sets. Receiver operating characteristic (ROC) curves of the seven ML models applied to NHANES and KNHANES femoral neck and total femur bone mineral density (BMD). The area under the curve (AUC) values of individual classes are noted in the graphs. KNANES: Korean National Health and Nutrition Examination Survey; KNN: k-nearest neighbor; LGBM: light gradient boosting machine; MLP: multilayer perceptron; NHANES: US National Health and Nutrition Examination Survey; SVM: support vector machine.

**Figure 4:**
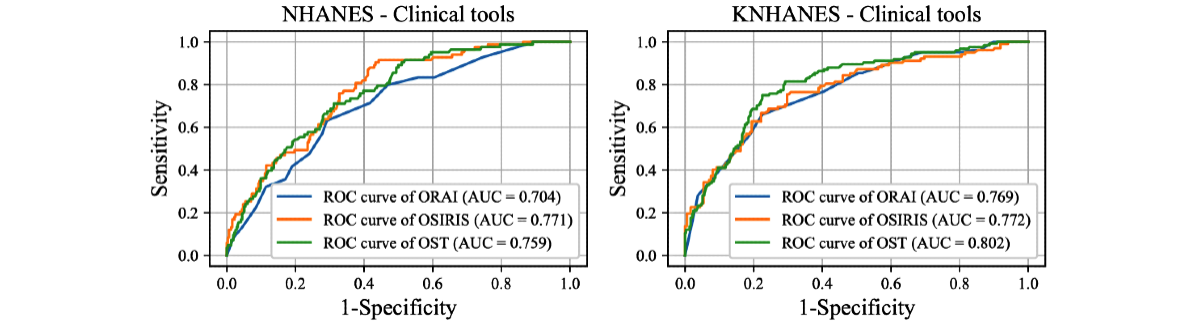
Performance of clinical osteoporosis assessment tools. AUC: area under the curve; KNHANES: Korean National Health and Nutrition Examination Survey; NHANES: US National Health and Nutrition Examination Survey; ORAI: Osteoporosis Risk Assessment Instrument; OSIRIS: Osteoporosis Index of Risk; OST: Osteoporosis Self-assessment Tool; ROC: receiver operating characteristic.

The receiver operating characteristic curves and corresponding AUC values of the optimized DL and ML models are presented in Figure 2 and Figure 3, respectively. The results were averaged across five-fold cross-validation. The DL model using NHANES exhibited a lower microaveraged AUC for the femoral neck BMD data set than for the total femur BMD data set (Table 2). Regarding individual classes, the classification of osteoporosis exhibited a higher AUC compared to the osteopenia and normal classes in the NHANES data set (Table 2). Similarly, for KNHANES, we achieved a higher AUC for the total femur data set than for the femoral neck BMD data set (Table 2). Additionally, the AUC for classifying osteoporosis was the highest in the KNHANES data set. Some of the ML models exhibited slightly lower performance than the DL models (Figure 3). Logistic regression exhibited the highest AUC values for all four data sets. The LGBM classifier and MLP exhibited the second-highest AUC values for the NHANES femoral neck BMD (Figure 3, Table 2). However, two ML models, namely decision trees and KNN, exhibited significantly lower performance than the other ML or DL models. Comparisons with conventional clinical risk assessment tools such as OST, ORAI, and OSIRIS were also performed (Figure 4). OSIRIS exhibited the highest AUC among the three clinical tools (0.771). The AUC of OST was 0.759 and that of ORAI was 0.704 for NHANES, indicating that the developed DL models outperformed the conventional osteoporosis assessment tools.

**Table 2:** Performance of deep-learning and machine-learning models.

Algorithm	AUC^a^ (95% CI)			
	NHANES^b^		KNHANES^c^	
	Femoral neck	Total femur	Femoral neck	Total femur
Deep learning (microaverage)	0.851 (0.844-0.858)	0.922 (0.916-0.928)	0.827 (0.821-0.833)	0.912 (0.898-0.927)
Deep learning (osteoporosis)	0.866 (0.842-0.890)	0.915 (0.898-0.931)	0.874 (0.858-0.890)	0.898 (0.884-0.911)
Deep learning (osteopenia)	0.719 (0.707-0.730)	0.784 (0.763-0.805)	0.688 (0.679-0.698)	0.782 (0.751-0.812)
Deep learning (normal)	0.783 (0.776-0.789)	0.827 (0.809-0.846)	0.823 (0.815-0.832)	0.822 (0.795-0.849)
Nonlinear SVM^d^	0.800 (0.761-0.838)	0.848 (0.791-0.906)	0.785 (0.775-0.795)	0.895 (0.888-0.902)
Extra trees	0.807 (0.802-0.812)	0.893 (0.883-0.903)	0.769 (0.761-0.777)	0.885 (0.882-0.887)
Logistic regression	0.827 (0.803-0.850)	0.900 (0.868-0.932)	0.794 (0.781-0.807)	0.905 (0.899-0.910)
KNN^e^	0.770 (0.763-0.778)	0.866 (0.855-0.878)	0.717 (0.711-0.724)	0.846 (0.837-0.855)
LGBM^f^ classifier	0.810 (0.804-0.821)	0.900 (0.899-0.918)	0.750 (0.745-0.759)	0.900 (0.900-0.905)
MLP^g^	0.819 (0.806-0.831)	0.902 (0.897-0.907)	0.766 (0.754-0.779)	0.869 (0.860-0.879)
Decision trees	0.668 (0.655-0.681)	0.761 (0.749-0.773)	0.649 (0.642-0.655)	0.755 (0.747-0.762)

^a^AUC: area under the curve.

^b^NHANES: US National Health and Nutrition Examination Survey.

^c^KNHANES: Korean National Health and Nutrition Examination Survey.

^d^SVM: support vector machine.

^e^KNN: k-nearest neighbor.

^f^LGBM: light gradient boosting machine.

^g^MLP: multilayer perceptron.

### Risk Factors and Individualized Explanations of Feature Contributions

The contribution rankings calculated using LIME for NHANES and KNHANES data are presented in Table 3 and Table 4, respectively. The results using Boruta and LASSO are presented in Multimedia Appendices 1-4. Based on the feature contribution rankings for NHANES and KNHANES, the high-ranked features were similar between the DL and ML models. These features include respondent sex, age, BMI, arm circumference, and prevalence of obesity. Other features also ranked in high positions, including economic status features such as occupation and family poverty income ratio (PIR), alkaline phosphatase, uric acid, depression, arthritis, and several nutrients (vitamin A, carotene, vitamin C, and vitamin D).

**Figure 5:**
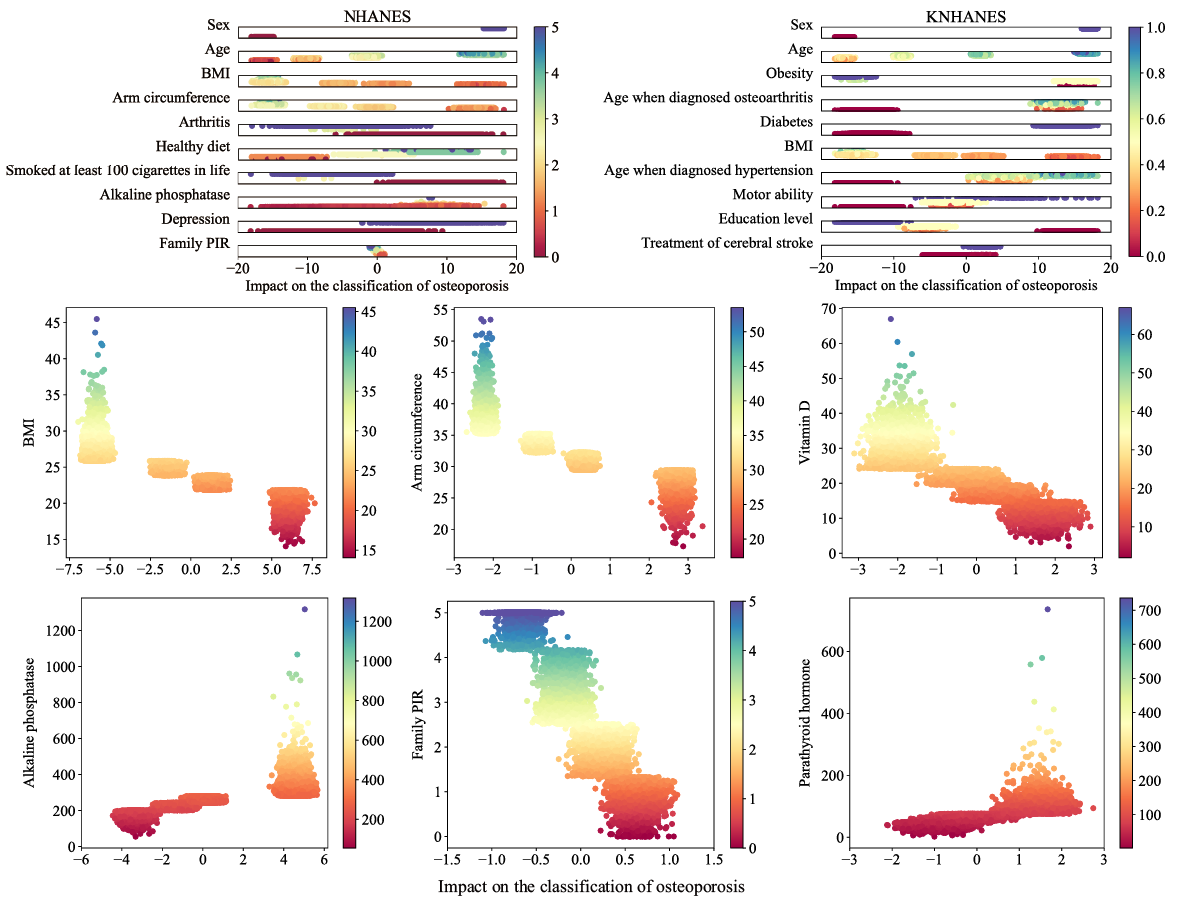
Impact of the top 10 features and 6 features in particular on the classification of osteoporosis. Visualized feature contribution values for the classification of osteoporosis using local interpretable model-agnostic explanations and deep-learning models. Each dot corresponds to one individual respondent, and different colors are used to represent feature magnitudes and categories. The magnitudes of both the numerical and categorical feature values are normalized in the range of 0 to 1 for comparison. The x-axis represents the impact value in percentage and the dots on the right tend to increase the probability of being included in the osteoporosis group. Impact graphs of the top 10 features of NHANES and KNHANES with femoral neck and total femur BMD integrated are shown on the top two graphs. The below six graphs show the impact values of BMI, arm circumference, vitamin D, alkaline phosphatase, family PIR, and parathyroid hormone presented with their original values on the y-axis. KNHANES: Korean National Health and Nutrition Examination Survey; NHANES: US National Health and Nutrition Examination Survey; PIR: poverty income ratio.

Figure 5 presents a visualization of the impact of the top 10 features according to the integrated contribution rankings of the femoral neck and total femur BMD. Each dot represents an individual respondent. Each feature’s values or categories were normalized and are expressed using different colors. For example, the smaller the BMI value, the more it contributes to the diagnosis of osteoporosis. The individual features and their relationships with the diagnosis of osteoporosis with nonnormalized values are also presented in Figure 5. For example, the graphs of BMI, arm circumference, vitamin D, and family PIR indicate that small values of these features contribute to osteoporosis diagnosis. In contrast, large values of alkaline phosphatase and parathyroid hormone increase the probability of diagnosis in the osteoporosis group.

**Figure 6:**
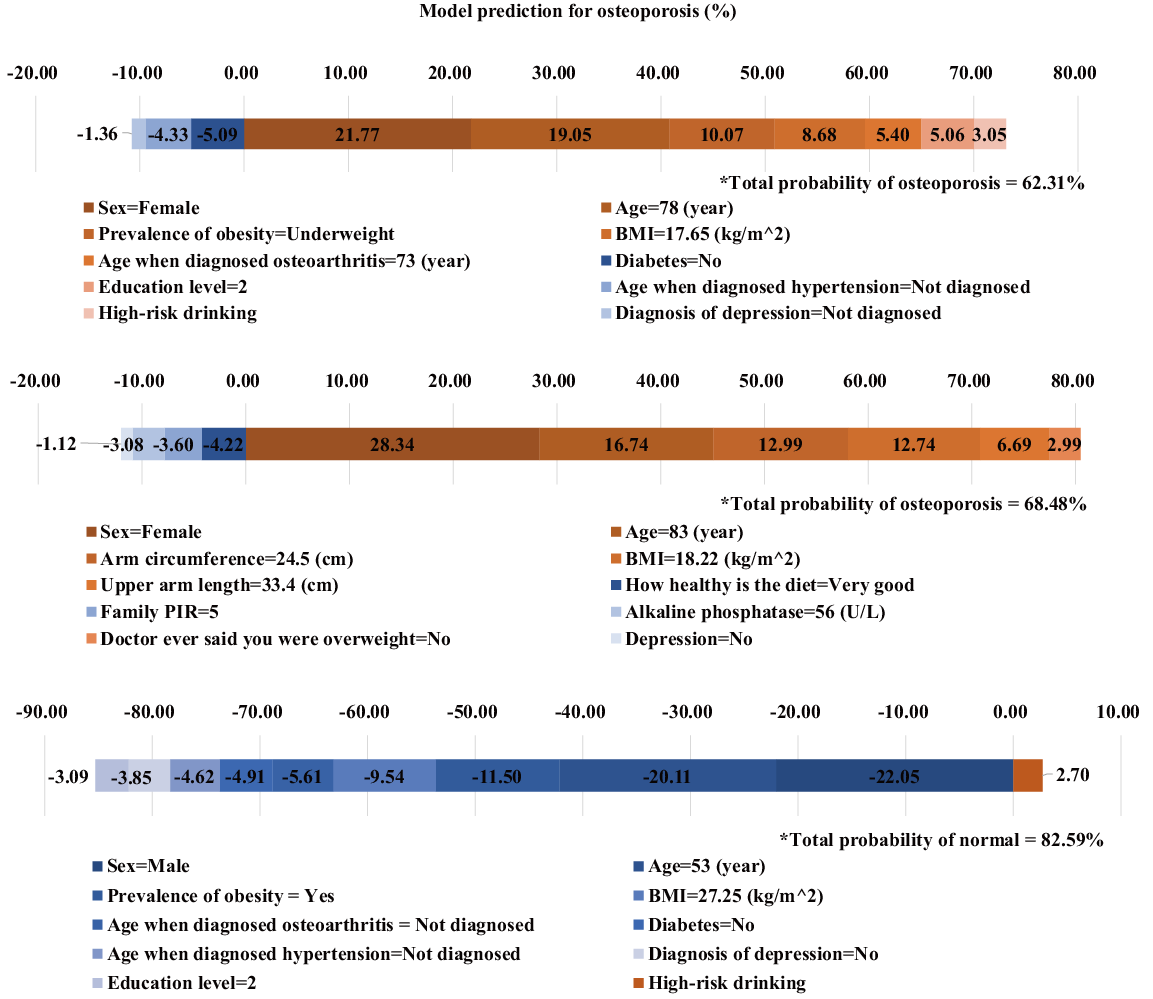
Individualized risk assessment for osteoporosis with feature integrated contributions from the explainable artificial intelligence model. Individualized osteoporosis risk predictions and the contributions of the top 10 features are shown in the graphs. The features in the legend are written from left to right in order of their absolute contribution values. Three representative respondents were selected and the properties of the top 10 features and their positive or negative impact on the probability of being diagnosed are presented with color bar graphs. A certain value of a feature with a positive impact value tends to increase the probability of model prediction for osteoporosis. In contrast, the impact value of a feature with negative impact tends to reduce the probability of model prediction for osteoporosis. For the first sample, the model predicts a probability of osteoporosis of 62.31% and for the second sample, it predicts a probability of 68.48%. The third sample is predicted as normal with a probability of 82.59%. “Education level” is classified based on the highest level of school the respondent completed, where class 1 represents elementary school, class 2 represents middle or high school, and class 3 represents college or above. “How healthy is the diet” is divided into five classes of “Excellent,” “Very good,” “Good,” “Fair,” and “Poor.” PIR: poverty income ratio.

Figure 6 presents the feature contributions to individual samples of respondents diagnosed with osteoporosis. The impact of each feature value on classifying these respondents into the osteoporosis group is represented in the graph. When comparing these three respondents, female sex increases the probability of being classified into the osteoporosis group. Additionally, the lower the BMI, the more likely an individual is to be diagnosed with osteoporosis. The explainable DL model for these individuals indicates that respondents with osteoporosis tend to have a low BMI and small body size. In terms of prophylactic use of risk factors, analysis results in younger respondents are most appropriate. Therefore, we present the analysis results for the age group of 50-60 years in Multimedia Appendices 5-8.

**Table 3:** Ranking of top 20 features from the deep-learning model and local interpretable model-agnostic explanations in the US National Health and Nutrition Examination Survey (NHANES).

Rank of NHANES	Femoral neck			Total femur	
	Description of features	Coefficient	Description of features		Coefficient
1	Sex	234.15	Sex		188.91
2	Age	171.67	Age		77.05
3	BMI (kg/m^2^)	115.66	Arm circumference (cm)		60.84
4	Arm circumference (cm)	108.35	BMI (kg/m^2^)		59.49
5	Doctor ever said you had arthritis	68.07	Doctor ever said you were overweight		37.31
6	Upper arm length (cm)	60.80	Upper arm length (cm)		30.45
7	Smoked at least 100 cigarettes in life	43.30	How healthy is the diet		20.29
8	Doctor ever confirmed you had high blood pressure	41.69	Alkaline phosphatase (U/L)		18.92
9	Doctor confirmed you have diabetes	39.18	Depression		18.87
10	Upper leg length (cm)	37.27	Family PIR^a^		17.33
11	Shortness of breath on stairs/inclines	35.47	Smoked at least 100 cigarettes in life		14.40
12	How healthy is the diet	30.09	General health condition		13.90
13	Family PIR	24.97	How often have urinary leakage		12.31
14	Doctor ever said you were overweight	22.42	Doctor confirmed you have diabetes		12.13
15	Monocyte number	22.18	Ever had pain or discomfort in chest		12.00
16	Alkaline phosphatase (U/L)	21.99	Age at heaviest weight		11.35
17	Potassium (mmol/L)	21.84	Doctor ever confirmed you had high blood pressure		11.12
18	LDL^b^-cholesterol, Friedewald (mg/dL)	21.82	Uric acid (mg/dL)		10.47
19	Uric acid (mg/dL)	20.56	Shortness of breath on stairs/inclines		9.45
20	Depression	16.98	Glucose (mg/dL)		8.68

^a^PIR: poverty income ratio.

^b^LDL: low-density lipoprotein.

**Table 4:** Ranking of top 20 features using the deep-learning model and local interpretable model-agnostic explanations in the Korean National Health and Nutrition Examination Survey (KNHANES).

Rank of KNHANES	Femoral neck			Total femur	
	Description of features	Coefficient	Description of features		Coefficient
1	Sex	596.16	Sex		108.62
2	Age	354.75	Age		106.44
3	Prevalence of obesity	227.33	Prevalence of obesity		79.23
4	Age when diagnosed with osteoarthritis	188.29	Marital status		65.44
5	Diabetes	166.55	Treatment of hepatitis B		55.64
6	BMI (kg/m^2^)	147.76	BMI (kg/m^2^)		52.58
7	Age when diagnosed with hypertension	144.57	Age when diagnosed with osteoarthritis		44.56
8	High-risk drinking	133.87	Motor ability		38.27
9	Education level	122.13	Presence of depression		37.48
10	Diagnosis of depression	116.04	Age when diagnosed with hypertension		34.67
11	Treatment of thyroid disease	112.91	Treatment of cerebral stroke		33.70
12	Age when diagnosed with angina	109.09	Age when diagnosed with myocardial infarction		33.68
13	Alkaline phosphatase (IU/L)	94.27	Presence of breast cancer		32.73
14	Treatment of cerebral stroke	92.46	Diagnosis of dyslipidemia		31.12
15	Menopause	92.10	Physical activity restriction		28.70
16	Diagnosis of dyslipidemia	88.83	Marital status		28.32
17	Occupation	88.02	Alkaline phosphatase (IU/L)		23.72
18	Diagnosis of colorectal cancer	85.92	Diagnosis of colorectal cancer		23.01
19	Presence of myocardial infarction or angina	83.92	High-strength physical activity		22.99
20	Diagnosis of rheumatoid arthritis	81.06	Middle-strength physical activity		22.93

## Discussion

### Principal Results

In this study, the XAI technique was applied to a DL model for osteoporosis risk screening using nationally representative samples from the NHANES and KNHANES data sets. Our DL model outperformed existing ML models, regression analysis, and clinical tools. Subsequently, we performed feature analysis by applying LIME to our DL model. The feature analysis results from LIME reconfirmed previously known features associated with osteoporosis risk, such as sex, age, and factors related to weight (eg, BMI, diagnosis of overweight and obesity). However, unexpected features that are easily ignored in clinical practice were also identified. Furthermore, our model provides individualized risk assessments for osteoporosis with an explanation of feature contributions.

To the best of our knowledge, this is the first study to use a DL model on medical big data to classify osteoporosis and LIME on the DL classifier and to select the most influential features. Previous studies have presented an application of DL or ML models to classify osteoporosis, estimate BMD, and predict osteoporotic fractures. However, previous studies utilizing DL have mainly focused on analyzing image data such as computed tomography [31-34] or X-ray images [35-39]. Most researchers have attempted to use ML models when analyzing tabular data. Additionally, some previous studies that examined important features related to osteoporosis with tabular data attempted to perform statistical analysis or ML based on statistical methods, including a Poisson regression model [40] and multivariate logistic regression [41] for feature selection. Related ML methods include correlation-based feature selection [42], backward elimination [43,44], LASSO applied to logistic regression models [45], use of Gini impurity on a gradient-boosting ML model [46], and outputs of interpretable ML models [47]. Only a few studies have adopted XAI techniques such as the Shapley additive explanation (SHAP), which is confined to ML classifiers. For example, Shim et al [44] used backward elimination to filter out features by considering their contribution to the outputs of a logistic regression model, and Chen et al [47] developed a hybrid model consisting of extreme gradient boosting (XGBoost) and an MLP, where the output values from XGBoost were used for feature selection. Another study that applied SHAP to select the most important features related to osteoporosis was conducted based on XGBoost, which is an ML model [48]. Similarly, Tanphiriyakun et al [49] trained seven ML models to predict the BMD response after the treatment of osteoporosis and analyzed the variable contribution using SHAP. Another study applied feature selection for a minority class method to ML models, KNN and random forest, to select useful information from the balance data [50].

Apart from these previous studies, we developed a DL classifier for screening osteoporosis using cross-sectional data and arranged features in order of importance for classifying respondents into osteoporosis groups using tabular LIME, which is an XAI technique. The strong performance of our model, as evaluated using AUC values, suggests that the DL classifier is also applicable to cross-sectional data analysis and osteoporosis diagnosis. Furthermore, concordance between the features selected through LIME and existing clinical risk factors indicated that the DL model utilized clinically significant features to classify respondents with osteoporosis. Despite the common concern that DL models are black-box models whose complexity makes it challenging to explain the decision-making process, our study demonstrates that interpretability can be achieved even for nonimage data if appropriate explanation techniques are adopted.

Existing studies support the major contributing features identified by LIME and their relationship to osteoporosis. Sex and age are widely known risk factors for osteoporosis, which coincides with several studies suggesting that female sex and low body weight are key risk factors for osteoporosis [51]. Reports on sex discrepancy can also be found for NHANES. For example, in the NHANES 2005-2006 data set, men had a lower prevalence of 30% in the osteopenia group and 2% in the osteoporosis group for femoral neck BMD compared to women with a prevalence of 49% in the osteopenia group and 10% in the osteoporosis group [52]. Other features worth noting for the NHANES and KNHANES data sets are economic status features, including occupation, family PIR, and house ownership. Some studies have observed that postmenopausal women in poverty or non-Hispanic white, Black, and Asian adults with low socioeconomic status have a lower BMD and higher risk of osteoporosis [53,54]. Measurements of alkaline phosphatase and uric acid were also included among the high-ranking features. It is known that patients with osteoporosis exhibit a high level of alkaline phosphatase [55] and patients with high uric acid levels tend to have lower BMD levels [56].

Generally, obesity has been considered to be a protective feature against osteoporosis by providing mechanical stimuli to the bones [57]. Our model indicates that several obesity-associated variables, including BMI, low-density lipoprotein cholesterol, triglycerides, arm circumference, prevalence of obesity, and dyslipidemia diagnosis, are significant features for osteoporosis and exhibit unified trends. In particular, for NHANES, arm circumference emerged as a high-ranking contributing feature. Research on the population living in the southern region of Stockholm indicated that among several anthropometric measurements, a small arm circumference was a stronger risk factor for osteoporosis than a low body weight [58]. Although recent mechanical, biochemical, and hormonal evidence has indicated an association between adipose tissue and deteriorating effects on the microarchitecture of the bone [59], our model identified linear trends for obesity and BMD.

Psychosocial behavior is often ignored as a risk factor for osteoporosis. However, our DL model indicates that depression-associated variables are significant risk factors. This may be associated with decreased physical activity, which is essential for maintaining bone strength, and negative effects of antidepressant medications on bone metabolism, including selective serotonin reuptake inhibitors [60]. Both arthritis and osteoporosis exhibit similar clinical features in terms of symptoms, treatment, and prognosis. However, they are different diseases with different etiologies. A potential role of systemic inflammatory diseases such as rheumatoid arthritis and ankylosing spondylitis in the development of osteoporosis has been proposed [61]. Additionally, several nutrients, including carotene and vitamins A, C, and D, ranked as significant features. Excluding vitamin D, the other nutrients have mainly been considered to be associated with deficient states. However, our model indicates the clinical significance of vitamin D. These diseases and nutrients should not be overlooked, and further research and consideration in clinical practice are required.

### Limitations

There are several limitations of this study that need to be addressed. First, the NHANES and KNHANES data sets utilized in this study were derived from cross-sectional surveys that collected data from a population at one specific point in time. Therefore, this study was confined to predicting the risk of osteoporosis at a specific point in time and we could not generate predictions for future occurrences. The DL model trained on cross-sectional data sets is limited to estimating the current osteoporosis status unless data sets with the label of future osteoporosis status, such as longitudinal cohort data, are utilized. Therefore, we plan to investigate training the DL model with longitudinal cohort data to predict the future risk of osteoporosis in follow-up work. Second, analysis of the BMD of the lumbar spine was not performed because the reference BMD for calculating the T-score for NHANES III did not contain lumbar spine BMD data. Moreover, both data sets lack information about the bone itself, such as bone turnover markers, with only BMD-related information provided. Given the clinical significance of osteoporosis, further use of data on the bone itself, including bone turnover markers, is expected to contribute to improving the model performance and reliability. Third, there was a high level of class imbalance in both the NHANES and KNHANES data sets, which occurred from a small number of respondents being diagnosed with osteoporosis. This imbalance may have caused the DL model to become biased toward the normal group with the highest number of respondents. We attempted applying techniques such as class weighting and the synthetic minority oversampling technique [62] to alleviate the class imbalance problem, but discontinued their use because they had a negative impact on model performance. Additionally, evaluation using the FRAX, which is one of the most widely used osteoporosis clinical assessment tools, was not performed in this study owing to significant differences between the formulae used in the FRAX for the United States and Korea, and the unavailability of an exact formula. Further, there exists a limitation of statistical analysis on the multicategorical features. Our DL model was trained using the original data format for most of the multicategorical features to minimize artificial factors unless unavoidable. However, some multicategorical features showed different results when subgroup analysis was used due to the nature of DL and LIME along with redundancy in features [63-65]. Finally, LIME is prone to instability because of randomness in the sampling step of its process [66]. Although we compared the selected features from LIME to commonly known clinical risk factors for osteoporosis and presented supporting references from previous research, it is still necessary to validate this process. Therefore, the analysis of the selected features from LIME should be performed carefully.

### Conclusions

In conclusion, we developed a DL model for classifying osteoporosis using NHANES and KNHANES data, which outperformed conventional clinical assessment tools and ML models. Additionally, we discussed important features selected based on XAI technology. This implies that DL can be fully applied to medical big data for the risk analysis of certain diseases. Furthermore, our model enables individualized osteoporosis risk assessment with an explanation for each feature’s contribution to model results. Despite the limitations noted above, the performance of the DL classifier and feature analysis results are noteworthy and demonstrate the potential for applying DL and XAI techniques to medical research, including cross-sectional studies. Utilizing cohort data for external validation of our DL model and training with the cohort data to predict the future risk of osteoporosis are considered as future work.

## Appendix

Multimedia Appendix 1 Ranking of top 20 features from NHANES using machine-learning models and Boruta.

Multimedia Appendix 2 Ranking of top 20 features from KNHANES using machine-learning models and Boruta.

Multimedia Appendix 3 Ranking of top 20 features from NHANES using machine-learning models and LASSO.

Multimedia Appendix 4 Ranking of top 20 features from KNHANES using machine-learning models and LASSO.

Multimedia Appendix 5 Descriptive statistics: Data distribution (number of respondents) in each osteoporosis class of the age group 50-60 years.

Multimedia Appendix 6 Receiver operating characteristic (ROC) curves of deep-learning models on NHANES and KNHANES respondents in the age group of 50-60 years.

Multimedia Appendix 7 Ranking of top 20 features from NHANES respondents in the age group of 50-60 years using the deep-learning model.

Multimedia Appendix 8 Ranking of top 20 features from KNHANES respondents in the age group of 50-60 years using the deep-learning model.

## Abbreviations

AUC: area under the curve
BMD: bone mineral density
DL: deep learning
DXA: dual-energy X-ray absorptiometry
FRAX: fracture risk assessment tool
KCDC: Korea Centers for Disease Control and Prevention
KNHANES: Korea National Health and Nutrition Examination Survey
KNN: k-nearest neighbors
LASSO: least absolute shrinkage and selection operator
LGBM: light gradient boosting machine
LIME: local interpretable model-agnostic explanations
ML: machine learning
MLP: multilayer perceptron
NHANES: National Health and Nutrition Examination Survey
ORAI: osteoporosis risk assessment instrument
OSIRIS: osteoporosis index of risk
OST: osteoporosis self-assessment tool
PHQ-9: Patient Health Questionnaire-9
PIR: poverty income ratio
SCORE: Simple Calculated Osteoporosis Risk Estimation
SHAP: Shapley additive explanation
XAI: explainable artificial intelligence
XGBoost: extreme gradient boosting
