# Supplementary material for: Interpretable Deep-Learning Approaches for Osteoporosis Risk Screening and Individualized Feature Analysis Using Large Population-Based Data: Model Development and Performance Evaluation
Source: J Med Internet Res. 2023 Jan 13;25:e40179. doi: 10.2196/40179 (PMC9883743; doi:10.2196/40179)
Supplement: Multimedia Appendix 1 [file jmir_v25i1e40179_app1.docx]

Multimedia Appendix 1. Ranking of top 20 features from NHANES using machine learning model and Boruta

| Rank of NHANES | Description of features | Feature importance | Rank of NHANES | Description of features | Feature importance |
| --- | --- | --- | --- | --- | --- |
| Femoral neck |  |  | Total femur |  |  |
|  |  |  |  |  |  |
| **1** | Arm circumference (cm) | 0.079476 | **1** | Arm circumference (cm) | 0.086883 |
| **2** | Age | 0.054586 | **2** | BMI (kg/m^2^) | 0.067067 |
| **3** | BMI^a^ (kg/m^2^) | 0.053028 | **3** | Sex | 0.051827 |
| **4** | Upper Arm Length (cm) | 0.039275 | **4** | Upper Arm Length (cm) | 0.047603 |
| **5** | Upper Leg Length (cm) | 0.026868 | **5** | Age | 0.041120 |
| **6** | Red cell count SI | 0.022290 | **6** | Upper Leg Length (cm) | 0.024306 |
| **7** | Age when heaviest weight | 0.019855 | **7** | Alanine aminotransferase (ALT) (U/L) | 0.022746 |
| **8** | Sex | 0.019622 | **8** | Family PIR | 0.021107 |
| **9** | Family PIR^b^ | 0.019506 | **9** | Red cell count SI | 0.020047 |
| **10** | Uric acid (mg/dL) | 0.018948 | **10** | Alkaline phosphatase (U/L) | 0.020025 |
| **11** | Hemoglobin (g/dL) | 0.018219 | **11** | Lymphocyte percent (%) | 0.019769 |
| **12** | Creatinine, urine (mg/dL) | 0.018117 | **12** | Albumin, urine (ug/mL) | 0.019072 |
| **13** | Alkaline phosphatase (U/L) | 0.017971 | **13** | Eosinophils percent (%) | 0.018410 |
| **14** | Albumin, urine (ug/mL) | 0.017597 | **14** | Creatinine, urine (mg/dL) | 0.017981 |
| **15** | Segmented neutrophils percent (%) | 0.017270 | **15** | Segmented neutrophils percent (%) | 0.017845 |
| **16** | Alanine aminotransferase (ALT) (U/L) | 0.017262 | **16** | Age when heaviest weight | 0.017706 |
| **17** | Creatinine (mg/dL) | 0.017157 | **17** | Hemoglobin (g/dL) | 0.017580 |
| **18** | LDL-Cholesterol, Friedewald (mg/dL) | 0.016603 | **18** | Uric acid (mg/dL) | 0.017113 |
| **19** | Lymphocyte percent (%) | 0.016273 | **19** | Vitamin A, RAE (mcg) | 0.016714 |
| **20** | Triglycerides (mg/dL) | 0.016159 | **20** | Triglycerides (mg/dL) | 0.015950 |

^a^BMI: body mass index

^b^PIR: poverty income ratio
