# Supplementary material for: Interpretable Deep-Learning Approaches for Osteoporosis Risk Screening and Individualized Feature Analysis Using Large Population-Based Data: Model Development and Performance Evaluation
Source: J Med Internet Res. 2023 Jan 13;25:e40179. doi: 10.2196/40179 (PMC9883743; doi:10.2196/40179)
Supplement: Multimedia Appendix 2 [file jmir_v25i1e40179_app2.docx]

Multimedia Appendix 2. Ranking of top 20 features from KNHANES using machine learning model and Boruta

| Rank of KNHANES | Description of features | Feature importance | Rank of KNHANES | Description of features | Feature importance |
| --- | --- | --- | --- | --- | --- |
| Femoral neck |  |  | Total femur |  |  |
|  |  |  |  |  |  |
| **1** | Age | 0.0747002 | **1** | Age | 0.091301 |
| **2** | BMI^a^ (kg/m^2^) | 0.0383821 | **2** | BMI (kg/m^2^) | 0.053671 |
| **3** | Menopause | 0.0289025 | **3** | Alkaline phosphatase (IU/L) | 0.029058 |
| **4** | Sex | 0.0281059 | **4** | Consumption of niacin per day (mg) | 0.025826 |
| **5** | Hemoglobin (g/dL) | 0.0236478 | **5** | Consumption of ash (g) | 0.024227 |
| **6** | Household income | 0.0235099 | **6** | Hemoglobin (g/dL) | 0.023483 |
| **7** | Alkaline phosphatase (IU/L) | 0.0233932 | **7** | Consumption of vitamin C (mg) | 0.021081 |
| **8** | Consumption of niacin (mg) | 0.0230189 | **8** | Education level | 0.019623 |
| **9** | Education level | 0.0226426 | **9** | Erythrocyte (Mil/uL) | 0.019589 |
| **10** | Consumption of ash (g) | 0.0218532 | **10** | Vitamin D from blood test (ng/mL) | 0.019211 |
| **11** | Erythrocyte (Mil/uL) | 0.0203883 | **11** | Parathyroid hormone (pg/mL) | 0.018832 |
| **12** | Consumption of vitamin A (μgRE) | 0.0191602 | **12** | Household income | 0.018732 |
| **13** | Consumption of carotene (μg) | 0.0183022 | **13** | Consumption of carotene (μg) | 0.017455 |
| **14** | Parathyroid hormone (pg/mL) | 0.0177911 | **14** | Thrombocyte (Thous/uL) | 0.017392 |
| **15** | Consumption of vitamin C (mg) | 0.0174658 | **15** | Consumption of vitamin A (μgRE) | 0.017381 |
| **16** | Vitamin D from blood test (ng/mL) | 0.0172473 | **16** | Menopause | 0.015939 |
| **17** | Leukocyte (Thous/uL) | 0.0168168 | **17** | Leukocyte (Thous/uL) | 0.015672 |
| **18** | Thrombocyte (Thous/uL) | 0.0167916 | **18** | HDL-Cholesterol (mg/dL) | 0.014618 |
| **19** | HDL-Cholesterol (mg/dL) | 0.0157828 | **19** | Sex | 0.013658 |
| **20** | Age when start drinking | 0.0155569 | **20** | Marital status | 0.013267 |

^a^BMI: body mass index
