# Supplementary material for: Interpretable Deep-Learning Approaches for Osteoporosis Risk Screening and Individualized Feature Analysis Using Large Population-Based Data: Model Development and Performance Evaluation
Source: J Med Internet Res. 2023 Jan 13;25:e40179. doi: 10.2196/40179 (PMC9883743; doi:10.2196/40179)
Supplement: Multimedia Appendix 3 [file jmir_v25i1e40179_app3.docx]

Multimedia Appendix 3. Ranking of top 20 features from NHANES using machine learning model and LASSO

| Rank of NHANES | Description of features | Feature importance | Rank of NHANES | Description of features | Feature importance |
| --- | --- | --- | --- | --- | --- |
| Femoral neck |  |  | Total femur |  |  |
|  |  |  |  |  |  |
| **1** | BMI^a^ (kg/m^2^) | -0·7592 | **1** | BMI (kg/m^2^) | -0·7909 |
| **2** | Arm circumference (cm) | -0·6728 | **2** | Arm circumference (cm) | -0·5163 |
| **3** | Age | 0·4853 | **3** | Alkaline phosphatase (U/L) | 0·3387 |
| **4** | Upper Arm Length (cm) | -0·4477 | **4** | Age | 0·3045 |
| **5** | Sex | 0·2263 | **5** | Sex | 0·2979 |
| **6** | Protein, total (g/dL) | -0·1985 | **6** | Upper Arm Length (cm) | -0·2848 |
| **7** | Segmented neutrophils percent (%) | 0·1323 | **7** | Age when heaviest weight | -0·0835 |
| **8** | Parents ever told had osteoporosis? | -0·0879 | **8** | How healthy is the diet | 0·0729 |
| **9** | Ever told you had a stroke | -0·0646 | **9** | Lymphocyte percent (%) | -0·0702 |
| **10** | Uric acid (mg/dL) | -0·0643 | **10** | Segmented neutrophils percent (%) | 0·0671 |
| **11** | How healthy is the diet | 0·0617 | **11** | Ever told you had a stroke | -0·0592 |
| **12** | Need special equipment to walk | -0·0594 | **12** | Need special equipment to walk | -0·0544 |
| **13** | Taking insulin now | -0·0593 | **13** | Require special healthcare equipment | -0·0499 |
| **14** | Ever told you had weak/failing kidneys | -0·0574 | **14** | Family PIR^b^ | -0·0446 |
| **15** | Upper Leg Length (cm) | -0·0562 | **15** | Ever receive blood transfusion | -0·0435 |
| **16** | Close relative had heart attack? | -0·0519 | **16** | General health condition | 0·0367 |
| **17** | Family PIR (Poverty Income Ratio) | -0·0434 | **17** | Hepatitis B core antibody | 0·0304 |
| **18** | Age when heaviest weight | -0·0406 | **18** | Taking insulin now | -0·0300 |
| **19** | Require special healthcare equipment | -0·0396 | **19** | Protein, total (g/dL) | -0·0291 |
| **20** | Ever receive blood transfusion | -0·0392 | **20** | Have head cold or chest cold | 0·0266 |

^a^BMI: body mass index

^b^PIR: poverty income ratio
