# Supplementary material for: Interpretable Deep-Learning Approaches for Osteoporosis Risk Screening and Individualized Feature Analysis Using Large Population-Based Data: Model Development and Performance Evaluation
Source: J Med Internet Res. 2023 Jan 13;25:e40179. doi: 10.2196/40179 (PMC9883743; doi:10.2196/40179)
Supplement: Multimedia Appendix 4 [file jmir_v25i1e40179_app4.docx]

Multimedia Appendix 4. Ranking of top 20 features from KNHANES using machine learning model and LASSO

| Rank of KNHANES | Description of features | Feature importance | Rank of KNHANES | Description of features | Feature importance |
| --- | --- | --- | --- | --- | --- |
| Femoral neck |  |  | Total femur |  |  |
|  |  |  |  |  |  |
| **1** | BMI^a^ (kg/m^2^) | -1·2399 | **1** | BMI (kg/m^2^) | -1·0830 |
| **2** | Age | 1·1212 | **2** | Age | 0·8428 |
| **3** | Alkaline phosphatase (IU/L) | 0·7659 | **3** | Alkaline phosphatase (IU/L) | 0·4519 |
| **4** | Sex | 0·4666 | **4** | Parathyroid hormone (pg/mL) | 0·3432 |
| **5** | Parathyroid hormone (pg/mL) | 0·2122 | **5** | Sex | 0·2378 |
| **6** | Consumption of vitamin A per day (μgRE) | -0·1989 | **6** | Marrital status | 0·1249 |
| **7** | Leukocyte (Thous/uL) | 0·1915 | **7** | Motor ability | 0·1107 |
| **8** | Vitamin D from blood test (ng/mL) | -0·1391 | **8** | Vitamin D from blood test (ng/mL) | -0·0989 |
| **9** | Treatment of cerebral stroke | 0·1372 | **9** | Leukocyte (Thous/uL) | 0·0962 |
| **10** | Consumption of ash (g) | -0·1315 | **10** | Consumption of ash (g) | -0·0932 |
| **11** | Education level | -0·1064 | **11** | Prevalence of obesity | -0·0552 |
| **12** | Motor ability | 0·0991 | **12** | Middle-strength physical activity | -0·0518 |
| **13** | Marrital status | 0·0977 | **13** | Age when diagnosed dyslipidemia | -0·0508 |
| **14** | Prevalence of obesity | -0·0941 | **14** | Prevalence of gastric cancer | 0·0505 |
| **15** | Thrombocyte (Thous/uL) | 0·0856 | **15** | Presence of depression | -0·0450 |
| **16** | Presence of myocardial infarction or angina | -0·0649 | **16** | House ownership | -0·0443 |
| **17** | Age when start drinking | -0·0588 | **17** | Diagnosis of thyroid disease | -0·0422 |
| **18** | Occupation | -0·0584 | **18** | Middle-strength physical activity | -0·0388 |
| **19** | House ownership | -0·0565 | **19** | Rate of thinking suicide | 0·0308 |
| **20** | Middle-strength physical activity | -0·0558 | **20** | Age when diagnosed diabetes | -0·0299 |

^a^BMI: body mass index
