# Supplementary material for: Interpretable Deep-Learning Approaches for Osteoporosis Risk Screening and Individualized Feature Analysis Using Large Population-Based Data: Model Development and Performance Evaluation
Source: J Med Internet Res. 2023 Jan 13;25:e40179. doi: 10.2196/40179 (PMC9883743; doi:10.2196/40179)
Supplement: Multimedia Appendix 5 [file jmir_v25i1e40179_app5.docx]

Multimedia Appendix 5. Descriptive statistics: The number of respondents data distributed in each osteoporosis class of age 50 to 60 group

|  | Normal | Osteopenia | Osteoporosis |
| --- | --- | --- | --- |
| NHANES – Femoral Neck | 1851 (68%) | 814 (30%) | 57 (2%) |
| NHANES – Total Femur | 2220 (82%) | 474 (17%) | 28 (1%) |
| KNHANES – Femoral Neck | 1802 (57%) | 1299 (41%) | 88 (2%) |
| KNHANES – Total Femur | 2826 (89%) | 356 (11%) | 7 (0%) |
