# Supplementary figures and images for: Interpretable Deep-Learning Approaches for Osteoporosis Risk Screening and Individualized Feature Analysis Using Large Population-Based Data: Model Development and Performance Evaluation
Source: J Med Internet Res. 2023 Jan 13;25:e40179. doi: 10.2196/40179 (PMC9883743; doi:10.2196/40179)

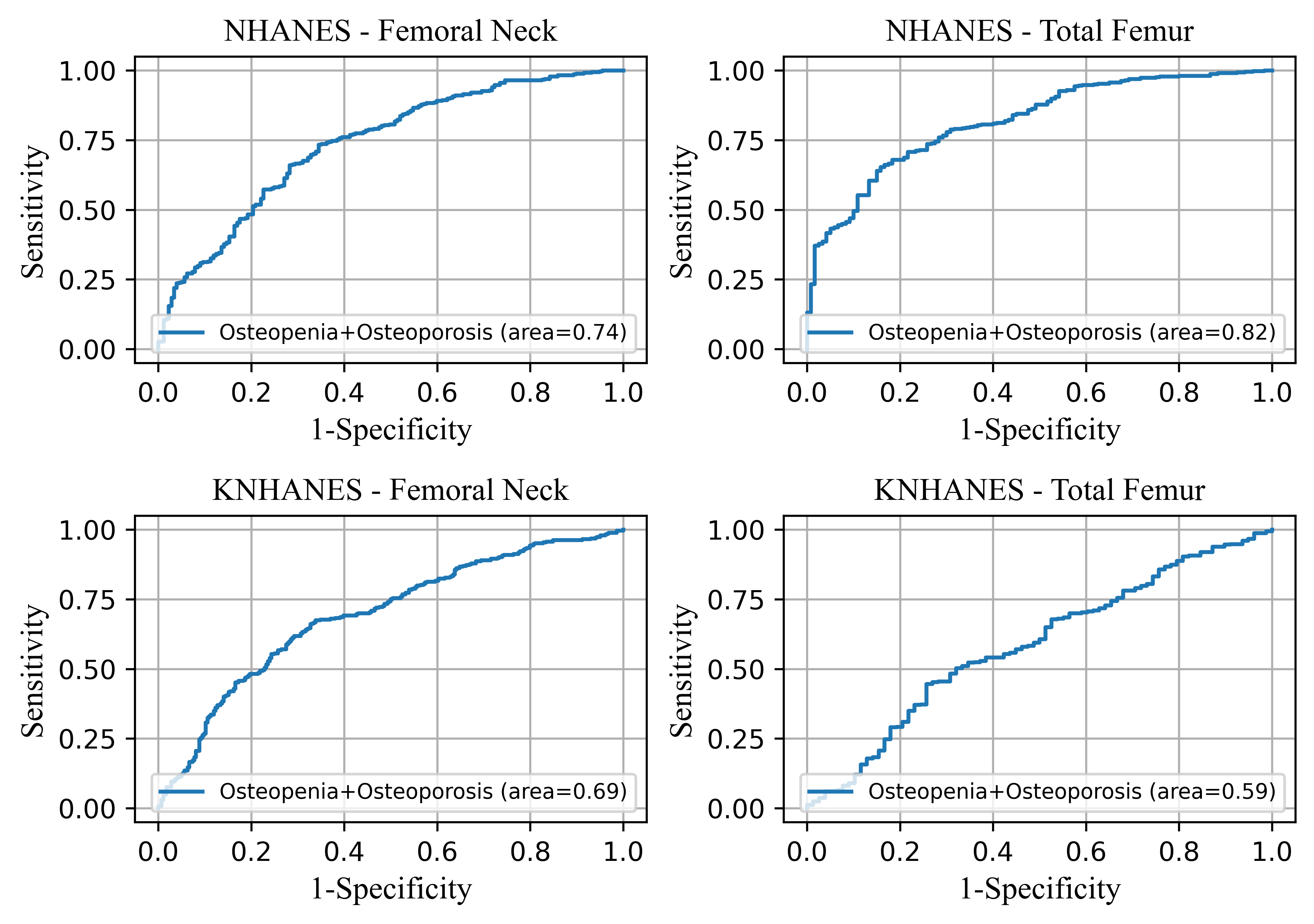

Supplement: Multimedia Appendix 6 [file jmir_v25i1e40179_app6.png]
