# Supplementary material for: Interpretable Deep-Learning Approaches for Osteoporosis Risk Screening and Individualized Feature Analysis Using Large Population-Based Data: Model Development and Performance Evaluation
Source: J Med Internet Res. 2023 Jan 13;25:e40179. doi: 10.2196/40179 (PMC9883743; doi:10.2196/40179)
Supplement: Multimedia Appendix 7 [file jmir_v25i1e40179_app7.docx]

Multimedia Appendix 7. Ranking of top 20 features from NHANES of age 50 to 60 group using DL model

| Rank of NHANES | Description of features | Coefficient | Rank of NHANES | Description of features | Coefficient |
| --- | --- | --- | --- | --- | --- |
| Femoral neck |  |  | Total femur |  |  |
|  |  |  |  |  |  |
| **1** | Sex | 442.52 | **1** | Sex | 845.56 |
| **2** | Age | 357.91 | **2** | Age | 330.46 |
| **3** | BMI^a^ (kg/m^2^) | 250.43 | **3** | BMI^a^ (kg/m^2^) | 290.03 |
| **4** | Arm circumference (cm) | 212.41 | **4** | Arm circumference (cm) | 250.00 |
| **5** | Upper Arm Length (cm) | 114.56 | **5** | Alkaline phosphatase (U/L) | 149.46 |
| **6** | Alkaline phosphatase (U/L) | 83.88 | **6** | Upper Arm Length (cm) | 128.18 |
| **7** | Ever told you had high blood pressure | 72.31 | **7** | Doctor ever said you were overweight | 111.60 |
| **8** | Doctor ever said you had arthritis | 68.51 | **8** | Smoked at least 100 cigarettes in life | 79.93 |
| **9** | Upper Leg Length (cm) | 63.93 | **9** | Family PIR^b^ | 71.32 |
| **10** | Shortness of breath on stairs/inclines | 62.65 | **10** | How healthy is the diet | 60.63 |
| **11** | Doctor told you have diabetes | 56.80 | **11** | General health condition | 56.75 |
| **12** | Family PIR^b^ | 44.98 | **12** | Ever had pain or discomfort in chest | 48.13 |
| **13** | Close relative had diabetes? | 42.11 | **13** | Doctor told you have diabetes | 41.99 |
| **14** | Potassium (mmol/L) | 42.02 | **14** | Uric acid (mg/dL) | 40.81 |
| **15** | Uric acid (mg/dL) | 40.56 | **15** | Age when heaviest weight | 40.30 |
| **16** | How healthy is the diet | 40.20 | **16** | Depression | 38.37 |
| **17** | Smoked at least 100 cigarettes in life | 38.34 | **17** | Triglycerides (mg/dL) | 36.47 |
| **18** | Sodium (mmol/L) | 36.83 | **18** | Lymphocyte percent (%) | 35.77 |
| **19** | LDL-Cholesterol, Friedewald (mg/dL) | 36.31 | **19** | Ever told you had high blood pressure | 34.61 |
| **20** | Doctor ever said you were overweight | 36.05 | **20** | Glucose (mg/dL) | 33.16 |

^a^BMI: body mass index

^b^PIR: poverty income ratio
