# Supplementary material for: Interpretable Deep-Learning Approaches for Osteoporosis Risk Screening and Individualized Feature Analysis Using Large Population-Based Data: Model Development and Performance Evaluation
Source: J Med Internet Res. 2023 Jan 13;25:e40179. doi: 10.2196/40179 (PMC9883743; doi:10.2196/40179)
Supplement: Multimedia Appendix 8 [file jmir_v25i1e40179_app8.docx]

Multimedia Appendix 8. Ranking of top 20 features from KNHANES of age 50 to 60 group using DL model

| Rank of KNHANES | Description of features | Coefficient | Rank of KNHANES | Description of features | Coefficient |
| --- | --- | --- | --- | --- | --- |
| Femoral neck |  |  | Total femur |  |  |
|  |  |  |  |  |  |
| **1** | Sex | 711.40 | **1** | Sex | 526.90 |
| **2** | Prevalence of obesity | 341.10 | **2** | Treatment of hepatitis B | 509.98 |
| **3** | Age | 329.42 | **3** | Prevalence of obesity | 425.49 |
| **4** | Treatment of hepatitis B | 227.12 | **4** | Marital status | 423.29 |
| **5** | Age when diagnosed osteoarthritis | 190.64 | **5** | Age | 410.74 |
| **6** | BMI^a^ (kg/m^2^) | 187.94 | **6** | BMI^a^ (kg/m^2^) | 296.77 |
| **7** | Diagnosis of depression | 177.55 | **7** | Age when diagnosed osteoarthritis | 207.32 |
| **8** | Age when diagnosed hypertension | 166.58 | **8** | Prevalence of breast cancer | 199.59 |
| **9** | Diabetes | 156.80 | **9** | Prevalence of depression | 183.97 |
| **10** | Treatment of thyroid disease | 144.91 | **10** | Treatment of depression | 176.55 |
| **11** | Age when diagnosed angina | 140.43 | **11** | Parathyroid hormone (pg/mL) | 173.69 |
| **12** | Diagnosis of cirrhosis | 115.32 | **12** | Diagnosis of dyslipidemia | 170.92 |
| **13** | Treatment of cerebral stroke | 115.30 | **13** | Motor ability | 165.47 |
| **14** | Alkaline phosphatase (IU/L) | 114.33 | **14** | Age when diagnosed hypertension | 165.41 |
| **15** | Diagnosis of dyslipidemia | 112.12 | **15** | Age when diagnosed myocardial infarction | 144.76 |
| **16** | Age when diagnosed dyslipidemia | 107.29 | **16** | Prevalence of cirrhosis | 143.82 |
| **17** | Menopause | 107.23 | **17** | Prevalence of rheumatoid arthritis | 142.74 |
| **18** | Prevalence of stomach cancer | 107.02 | **18** | Age when diagnosed cirrhosis | 142.51 |
| **19** | Prevalence of hypertension | 104.85 | **19** | Prevalence of cancer | 138.52 |
| **20** | Diagnosis of osteoarthritis | 104.39 | **20** | Alkaline phosphatase (IU/L) | 138.20 |

^a^BMI: body mass index
